# On-Chip Cell Staining and Counting Platform for the Rapid Detection of Blood Cells in Cerebrospinal Fluid

**DOI:** 10.3390/s18041124

**Published:** 2018-04-07

**Authors:** Yujin Lee, Byeongyeon Kim, Sungyoung Choi

**Affiliations:** Department of Biomedical Engineering, Kyung Hee University, 1732 Deogyeong-daero, Giheung-gu, Yongin-si, Gyeonggi-do 17104, Korea; yujin_61@khu.ac.kr (Y.L.); kby921022@khu.ac.kr (B.K.)

**Keywords:** blood cell count, cerebrospinal fluid, microfluidics, on-chip sample preparation, portable cell counter, miniaturized microscopy

## Abstract

Counting blood cells in cerebrospinal fluid (CSF) is indispensable for diagnosing several pathological conditions in the central nervous system, such as meningitis, even though collecting CSF samples is invasive. Cell counting methods, such as hemocytometer chambers and flow cytometers, have been used for CSF cell counting, but they often lack the sensitivity to detect low blood cell numbers. They also depend on off-chip, manual sample preparation or require bulky, costly equipment, thereby limiting their clinical utility. Here, we present a portable cell counting platform for simple, rapid CSF cell counting that integrates a microfluidic cell counting chamber with a miniaturized microscope. The microfluidic chamber is designed not only to be a reagent container for on-chip cell staining but also to have a large control volume for accurate cell counting. The proposed microscope miniaturizes both bright-field and fluorescence microscopy with a simple optical setup and a custom cell-counting program, thereby allowing rapid and automated cell counting of nucleated white blood cells and non-nucleated red blood cells in fluorescence and bright-field images. Using these unique features, we successfully demonstrate the ability of our counting platform to measure low CSF cell counts without sample preparation.

## 1. Introduction

Counting blood cells in cerebrospinal fluid (CSF) is a clinical test to diagnose pathological conditions of the central nervous system (CNS) such as meningitis, multiple sclerosis, hemorrhages, and tumors [1,2,3,4,5]. CSF is a clear body fluid surrounding the brain and spine with a white blood cell (WBC) count of about 5 cells per microliter and no red blood cells (RBCs) under normal conditions. A significant increase in the WBC count can indicate infection or inflammation of the CNS or certain types of tumors (i.e., CNS and metastatic tumors). The presence of RBCs in CSF can be a sign of bleeding into CSF caused by aneurysm rupture, arteriovenous malformation, or head injury. Although collecting CSF samples is invasive, CSF cell counting is indispensable for diagnosing certain pathological conditions, especially in evaluating chronic meningitis [5].

Hemocytometer chambers are routinely used to count blood cells in a defined volume of CSF under a microscope [6]. Although this technique is straightforward and effective for counting cells, it requires laborious, time-consuming, and manual processes for cell staining and counting and often lacks the sensitivity and reliability to detect low numbers of blood cells in CSF, generally less than 100 WBCs per microliter in patients with viral meningitis [1]. The cumbersome counting method has been recently improved by incorporating image recognition algorithms to automate cell counting and cell-enrichment cartridges to improve counting sensitivity [7,8]. However, the additional off-chip sample preparation steps to fluorescently stain and enrich blood cells increase experimental workload and require well-trained operators. As an alternative to hemocytometer-based counting methods, flow cytometers enable automatic counting of CSF blood cells by using their impedance and light-scattering properties [9,10,11,12]; however, these technologies require bulky and costly equipment that can limit rapid and on-site testing of delicate CSF samples.

Considerable effort has been devoted to developing simple and portable imaging cytometry platforms that take advantages of microfluidics and simplified optical systems [13,14,15,16,17,18,19,20]. For instance, Moon et al. directly integrated a microfluidic chamber with an imaging sensor for continuous and lens-free counting of blood cells [17]. Im et al. developed a holographic microscopy on a smartphone for cancer diagnosis in resource-limited clinical settings [18]. Zhu et al. demonstrated a smartphone interface for imaging cytometry and integrated it with a microfluidic chamber to continuously count fluorescently-labeled blood cells [19]. Our group previously developed a portable fluorescence cell counter capable of performing on-chip staining and counting of somatic cells [20]. Although these platforms enable effective miniaturization of the readout optics, and simple and rapid testing of clinical samples, they often rely on off-chip, manual sample preparation that can increase experimental workload and cost, or lack the ability to perform both bright-field and fluorescence imaging that is often required to simultaneously count RBCs and WBCs. Consequently, a simple, sample preparation-free, and portable technology that can simultaneously enumerate RBCs and WBCs is still needed for CSF diagnostics.

**Figure 1:**
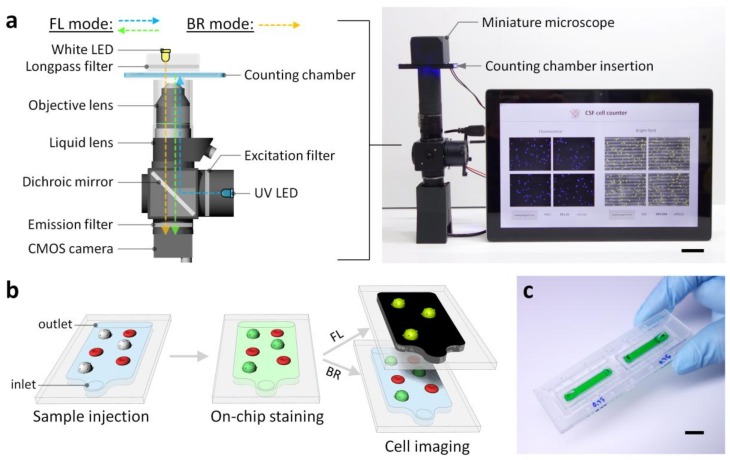
Portable platform for counting blood cells in CSF. (**a**) Schematic and photograph of the miniaturized bright-field and fluorescence microscopy which comprises a UV LED, an excitation filter (474 nm center wavelength), a dichroic mirror, a 20× objective lens, a liquid lens, an emission filter (525 nm center wavelength), a white LED, and a long-pass filter (500 nm cut-on wavelength). Note that the schematic is not to scale and used to describe the internal optical components. The custom Matlab-based graphic user interface enables automatic RBC and WBC counting from bright-field (BR) and fluorescence (FL) images. Scale bar, 3 cm. (**b**) Illustrations of the cell counting chamber and on-chip fluorescence staining process. As a CSF sample is loaded, the cell permeable dye deposited on the chamber bottom dissolves and fluorescently stains nucleated cells. (**c**) Photograph of the cell counting chamber filled with food dye for visualization. Scale bar, 1 cm.

Here, we present a comprehensive cell counting platform that allows on-chip staining of WBCs in minutes and in-situ cell counting of nucleated WBCs and non-nucleated RBCs in fluorescence and bright-field images (Figure 1). The platform comprises a microfluidic cell counting chamber with a stored, dried fluorescent nuclear stain that instantly dissolves in a sample fluid and stains target cells, and a miniaturized microscope with a light-adjustable lens that can rapidly capture in-focus bright-field and fluorescence cell images. In addition, a custom cell-counting program enables automatic cell counting. These unique features significantly improve blood cell counting in CSF by saving time and labor from sample injection to analysis without user intervention. We performed parametric studies of key parameters (i.e., dye concentration and incubation time) that can influence the performance of the cell counting platform. After optimizing the parameters, we tested the clinical utility of the platform by performing simple, rapid, portable, and automated counting of WBCs and RBCs in CSF.

## 2. Materials and Methods 

### 2.1. Materials and Sample Preparation

Canine blood samples were obtained from the Korea Animal Blood Bank with safety regulations. For WBC purification, whole blood was lysed with RBC lysis buffer (Biolegend, San Diego, CA, USA) and then washed twice with phosphate buffered saline (PBS) (Welgene, Inc., Gyeongsan-si, Korea) supplemented with 1% bovine serum albumin. RBCs and WBCs were then diluted with PBS to the desired concentration. Normal CSF samples with no detectable blood cells were purchased from Lee Biosolutions, Inc. (Jefferson City, MO, USA). A model of diseased CSF samples was prepared by spiking blood cells into the normal CSF samples. The resulting cell suspensions were pipetted into the microfluidic counting chamber. A cell-permeable nucleic acid dye, acridine orange (AO), was purchased from Sigma-Aldrich Corp. (St. Louis, MO, USA) to selectively stain WBCs. AO dye exhibits enhanced fluorescence when bound to DNA, which enables selective on-chip staining of WBCs without additional sample preparation steps such as membrane permeabilization and cell washing.

### 2.2. The Design and Fabrication of the Microfluidic Counting Chamber

**Figure 2:**
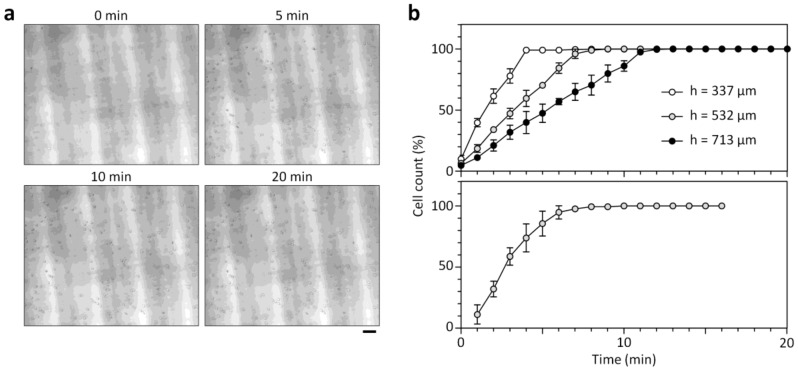
Blood cell sedimentation. (**a**) Successive microscopic images of settling RBCs in the 532-μm-high microfluidic counting chamber. All out-of-focus or blurry cells right after sample injection come into focus after 10 min. Scale bar, 50 μm. (**b**) (**top**) The critical time for complete RBC sedimentation depends on the chamber height, and is ≈7, 9 and 12.3 min for *h*_c_ = 337, 532, and 713 μm, respectively. (**bottom**) Settling kinetics of WBCs in the 532-μm-high counting chamber. Error bars: s.d. (*n* = 3).

The microfluidic chamber was designed to be 532 μm high, unless specified otherwise, which is about five times higher than a standard hemocytometer chamber. The increase in height allows accurate detection of low blood-cell concentrations. The chamber was fabricated by assembling an upper part that contains a microchannel with an inlet and outlet for sample injection and a flat bottom part that is coated with the fluorescent dye (Figure 1). The upper part of the chamber was printed with a stereolithography-based 3D printer (DWS systems Corp., Thiene, Italy) for rapid prototyping. The bottom part was the bottom layer of a disposable hemocytometer chip (Logos Biosystems, Inc., Anyang-si, Korea). The shadow and grid patterns in the background of the bright field images were attributed to the visible printing layers of 3D-printed microfluidic chambers (Figure 2), but did not affect cell counting due to the high contrast images of cells compared with the blur images of the patterns. The chamber clarity can be further improved by adopting mold-based fabrication methods such as injection molding instead of 3D printing. Before part assembly, the bottom part of the microfluidic chamber was mounted on a spin coater (Midas System Corp., Daejeon, Korea.), 2 μL of AO dye solution was applied in the center, and it was spin-coated at 1000 rpm for 30 s. The dye was dissolved in ethanol (Sigma-Aldrich) to rapidly evaporate the solvent and uniformly deposit the dye. The upper and bottom parts were then irreversibly bonded with liquid adhesive.

### 2.3. The Design and Fabrication of the Miniaturized Microscope

The miniaturized microscope was designed for both bright-field and fluorescence imaging, and fabricated by assembling a CMOS camera (FLIR, Inc., Victoria, British Columbia, Canada), a dichroic mirror (Semrock, Inc., Rochester, NY, USA), an excitation filter (Semrock) with a 474 nm center wavelength, an emission filter (Semrock) with a 525 nm center wavelength, a long-pass filter (Edmund Optics, Inc., NJ, USA) with a 500 nm cut-on wavelength, a liquid lens (Optotune, Inc., Zurich, Switzerland), a white LED (JENO Corp., Seoul, Korea), and a UV LED (LED Engin, Inc., San Jose, CA, USA) (Figure 1 and Figure S1). The housing for the optical components was printed with the 3D printer. The long-pass filter was placed between the white LED and microfluidic chamber to prevent the UV light from unintentionally illuminating a phosphor coated on the emitter of the white LED. Thus, this optical setup enables clear fluorescence imaging without a mechanical shutter. The liquid lens was used for rapid autofocusing during bright-field and fluorescence cell imaging, allowing for the rapid acquisition of multiple in-focus images. In addition, the incorporation of an electronic on–off switch enables easy transition between the bright-field and fluorescence imaging mode. The field of view (FOV) of the miniaturized microscope was 0.61 mm × 0.46 mm.

### 2.4. The Cell Counting Algorithm 

A custom Matlab-based graphic user interface was built for automatic blood cell counting. The program reads bright-field and fluorescence images taken in the same area and detects circular objects in the digital images based on the circle Hough transform algorithm to count cells (Figures S2 and S3). Briefly, the cell counting algorithm detects cells based on the radial symmetry and size of microscale objects. Since cell debris and clumps had a low degree of radial symmetry, and they were respectively smaller and larger than cells, cells could be successfully detected with a sensitivity threshold of 0.9, and a lower and upper size cut-off of 8.6 μm and 14.2 μm in diameter. The sensitivity threshold defines the radial symmetry of an object. As the threshold increases, the amount of rounded objects that can be detected decreases. WBCs were identified in a fluorescence image, and RBC counts were calculated by subtracting the WBC count from the total cell count obtained from a bright-field image. To calculate cell concentrations, the cell number counted in four different areas of each chamber were divided by the corresponding volume, 588 nL. The cell counts for both RBCs and WBCs measured by the counting program showed good agreement with the results determined by manual counting (98.71 ± 1.85% of accuracy, *n* = 40).

## 3. Results and Discussion

### 3.1. The Cell Counting Platform Design

The portable platform for CSF cell counting incorporates on-chip sample preparation and miniaturized integration of bright-field and fluorescence microscopy (Figure 1). Cells are counted by injecting a CSF sample into the 532-μm-high microfluidic counting chamber, which stores a nuclear staining dye deposited on the bottom (Figure 1b,c). The microfluidic chamber provides two major functions: a reagent container that enables on-chip cell staining to identify nucleated cells in situ and a large control volume for counting cells at low concentrations. The miniaturized microscope comprises a white LED with a broad spectrum and a 460 nm UV LED, thereby permitting both bright-field and fluorescence imaging (Figure 1a). The 500 nm long-pass filter prevents the UV light from unintentionally illuminating a phosphor coated on the emitter of the white LED, and thus ensures clear fluorescence imaging without a mechanical shutter (Figure S4). In addition, the ability of the microscope to automatically adjust focus with a liquid lens allows fast, in-focus bright-field, and fluorescence imaging at different focus levels. After the on-chip staining of WBCs, WBCs and RBCs are counted by capturing bright-field and fluorescence cell images and automatically counting fluorescent WBCs and non-fluorescent RBCs. The versatility and small footprint of the cell counting platform make it ideal for promptly counting blood cells in CSF samples, which are typically rare and easily degrade ex vivo [2].

### 3.2. Cell Sedimentation in Cell Counting Chambers

The microfluidic counting chamber is designed to count low cell concentrations due to the increased chamber height (*h*_c_) and the larger control volume than a standard hemocytometer chamber. Considering that cells need to settle down for in-focus imaging in the counting chamber higher than the focal depth of the miniaturized microscope, we first studied the sedimentation kinetics of blood cells injected into the chambers (Figure 2). As shown in Figure 2b, the time for the complete sedimentation (*t*_s_) of RBCs increases with *h*_c_, and was ≈7, 9, and 12.3 min for *h*_c_ = 337, 532, and 713 μm, respectively. Under test conditions *V*_c_ = 90 μm^3^ [21], *ρ*_c_ =1.09 g/mL [21], *ρ*_m_ = 1.04 g/mL, and *μ*_m_ = 1.05 cP, the gravitational settling velocity of RBCs is calculated to 0.92 μm/s, where *V*_c_ is the volume of RBCs, *ρ*_c_ is the density of RBCs, *ρ*_m_ is the buffer density and *μ*_m_ is the buffer viscosity. The resulting *t*_s_ is respectively estimated to ≈6.1, 9.6, and 12.9 min, which is in good agreement with the experimental results. We also obtained similar results with WBCs in the 532-μm-high counting chamber (Figure 2b). At low cell concentrations, a higher counting chamber can allow for accurate cell counting by enriching more cells on the chamber bottom. However, *h*_c_ cannot be too high because a long *t*_s_ prolongs the testing time. Using these results, we identified an optimum *h*_c_ of 532 μm, which improves the counting sensitivity fivefold compared to a standard hemocytometer with a height of 100 μm.

### 3.3. Optimization of On-Chip Cell Staining Protocols

**Figure 3:**
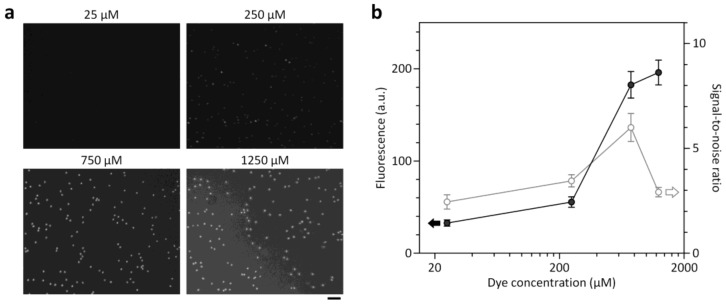
Effect of different dye concentrations on the fluorescence intensity of stained WBCs (signal) and background fluorescence (noise). (**a**) Fluorescence images of WBCs in the counting chamber at dye concentrations ranging from 25 to 1250 μM. Each micrograph was taken with the miniaturized microscope 10 min after introducing WBC samples. Scale bar, 50 μm. (**b**) The signal-to-noise ratio peaked at 750 μM, while the fluorescence intensity saturated at higher concentrations. Error bars: s.d. (*n* = 10).

**Figure 4:**
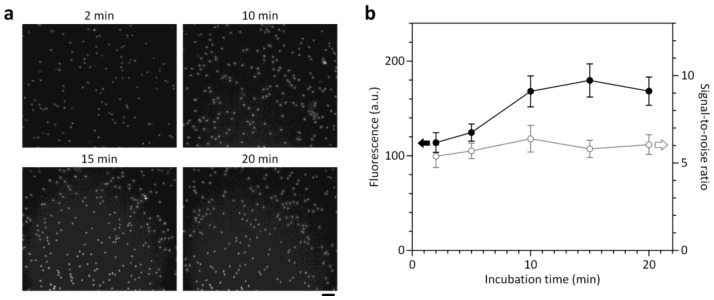
Kinetics of on-chip fluorescence cell staining. (**a**) Fluorescence images showing the time evolution of WBC staining in the counting chamber at a dye concentration of 750 μM. Scale bar, 50 μm. (**b**) Over time, the signal-to-noise ratio did not change significantly, while the fluorescence intensity of WBCs reached a plateau at 10 min. Error bars: s.d. (*n* = 10).

The ability for on-chip staining of target cells is central to developing a simple and user-friendly cell counting platform. To optimize on-chip cell staining protocols by maximizing target cell fluorescence (signal) while minimizing background level (noise), we performed a parametric study to determine optimum conditions for staining dye concentration (*c*_d_) and incubation time (*t*_i_). A nucleic acid-specific fluorescent dye, acridine orange, was used for on-chip staining of WBCs due to its high cell permeability and effective quantum yield characteristics that improve significantly when bound to DNA. The dye concentration stated is the concentration of the stock solution used for the coating process. Considering that most of the stock solution applied was discarded during the coating process and the fluorescence signals of cells stained in the chamber were relatively low, the actual concentration of AO dye dissolved in the chamber will be low enough not to affect the cells. The parametric study was performed by injecting intact WBCs at ≈10^3^ cells/μL into the cell counting chamber and taking fluorescence pictures using the miniaturized microscope. We first studied the effect of *c*_d_ on the fluorescence intensity of stained WBCs and the background fluorescence level (Figure 3). Although unbound dye had low fluorescence due to its low quantum yield, the background fluorescence level increases with *c*_d_ (Figure S5) and can affect the signal-to-noise ratio (SNR) of fluorescence images. As shown in Figure 3, the optimum *c*_d_ was 750 μM where fluorescently stained WBCs were clearly distinguished without significant background. Above the optimum *c*_d_, the fluorescence intensity of stained WBCs begins to saturate, while the background continues to increase, thereby impairing the SNR. We next investigated the kinetics of on-chip cell staining at *c*_d_ = 750 μM by monitoring the evolution of the fluorescence intensity of WBCs and background fluorescence over time (Figure 4). The fluorescence intensity of the cells increased for 10 min after sample loading and then reached a plateau. We obtained high SNRs (above 5.4) even at early time points (2–4 min). This result might be attributed to the high permeability of the dye through cell membrane and the low fluorescence background. As a result, we found optimized conditions (*c*_d_ = 750 μM and *t*_i_ = 10 min) to ensure clear identification of fluorescently stained cells and complete cell sedimentation.

### 3.4. Blood Cell Enumeration

**Figure 5:**
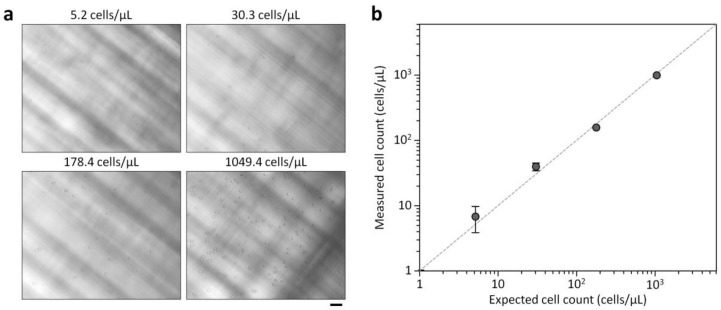
RBC counts measured with the cell counting platform and estimated with a standard hemocytometer. (**a**) Bright-field images of RBCs in the counting chamber at different cell concentrations. Scale bar, 50 μm. (**b**) On-chip RBC counts versus expected RBC counts from the hemocytometer, with the dotted line representing the unity slope. Error bars: s.d. (*n* = 3).

**Figure 6:**
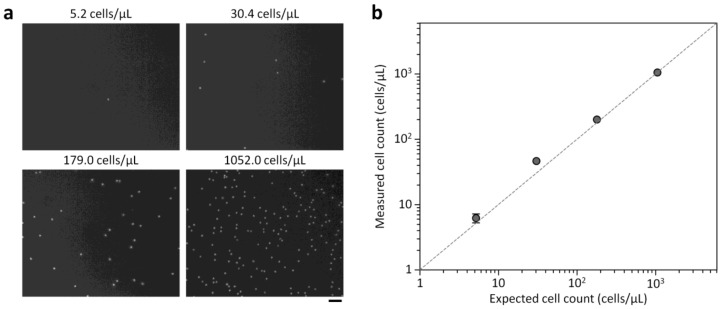
WBC counts measured with the cell counting platform and estimated with a standard hemocytometer. (**a**) Fluorescence images of stained WBCs in the counting chamber at different cell concentrations. Scale bar, 50 μm. (**b**) On-chip WBC counts versus expected WBC counts from the hemocytometer, with the dotted line representing the unity slope. Error bars: s.d. (*n* = 3).

Using the optimized conditions, we explored the ability of our platform to analyze cell counts as low as 5 cells/μL. RBCs and WBCs were counted by taking images with the miniaturized microscope and analyzing them with the custom Matlab program developed to automatically count microscopic objects. RBC and WBC samples were separately prepared in PBS for platform characterization. The expected cell concentrations were prepared by serially diluting a defined cell suspension (≈10^3^ cells/μL), which was measured with a conventional hemocytometer, and compared to the results obtained by the counting platform. As shown in Figure 5 and Figure 6, the cell counts measured by the counting platform closely matched the expected cell counts, showing a strong linear relationship down to 5.2 cells/μL with a regression slope of 0.9889 and an *R*^2^ of 0.9996 for WBC counts (*c*_wbc_) and a regression slope of 0.9424 and an *R*^2^ of 0.9996 for RBC counts (*c*_rbc_). These results indicate that the proposed platform can sensitively analyze low blood cell counts. In bacterial meningitis, WBC counts in CSF are typically higher than 1000 cells/μL, while the counts are usually less than 100 cells/μL with viral meningitis [1]. Bacterial meningitis is a serious disease requiring antibiotic therapy, while viral meningitis is less serious and tends to resolve without specific treatment [22]. Thus, diagnostic tests to distinguish between bacterial and viral meningitis are important to prevent unnecessary antibiotic use and decide proper treatment strategy. We note that the coefficient of variation (CV) for *c*_wbc_ = 5.2 cells/μL was 15.7%, while the CVs for other conditions ranged from 5.6 to 11.2%. The CV for *c*_rbc_ = 5.2 cells/μL was 43.3%, while the CVs for other conditions ranged from 0.8 to 13.8%. The performances of the counting platform was comparable to results found using automated hematology analyzers [9,11]. Since cell counting accuracy depends on the sample volume analyzed, it is important to enlarge the FOV of the miniaturized microscope to enhance cell counting accuracy. Adopting lensless holographic microscope systems with high resolution and large FOVs [23] might be a simple means of improving cell counting accuracy that retains the compactness and simplicity of the proposed counting platform.

**Figure 7:**
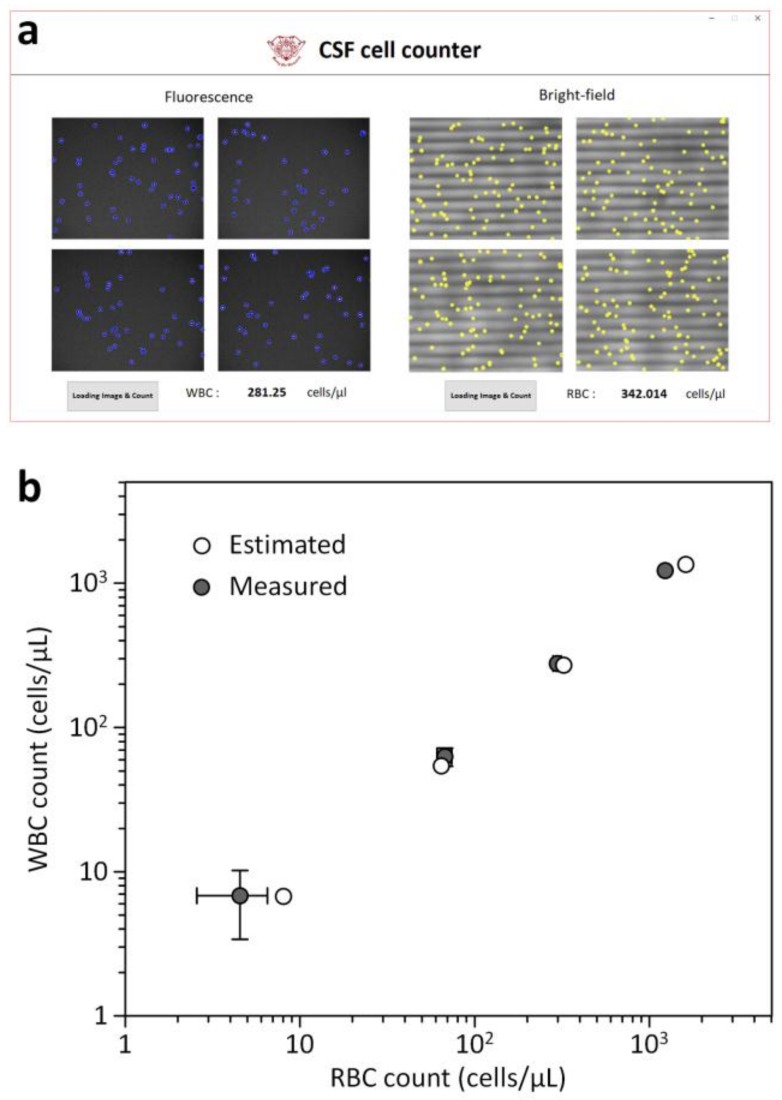
Blood cell counts in model CSF samples. (**a**) Custom Matlab GUI and cell counting program. Blood cells in bright-field and fluorescence images were overlaid with circle symbols for visualization. (**b**) The RBC and WBC counts of model CSF samples were automatically measured with the counting program and were highly correlated with the cell counts estimated with a standard hemocytometer.

We then tested the utility of the portable cell counting platform by using model CSF samples that contained both RBCs and WBCs in CSF. Figure 7 shows strong correlation between the expected and measured cell counts for both RBCs and WBCs (*R*^2^ ≥ 0.9996) in the concentration range of ≈4.5 cells/μL to ≈1.2 × 10^3^ cells/μL, indicating that our platform enables accurate analysis of WBCs and RBCs in CSF. Since blood cells gradually degrade in CSF [2], prompt testing is important to avoid false negative results. The advantages of the proposed platform, such as high portability, ease of operation, no need for sample preparation, and low sample volumes, make it suitable for rapid and simple analysis of blood cells in CSF. During the counting process of ≈10 min, there were no significant difference in the device performance between the PBS and CSF samples. We note that blood cells in CSF significantly degraded for a prolonged period of time (>60 min). These results support that the rapid detection of blood cells in CSF will be important for accurate diagnosis of diseases and conditions of the central nervous system. The proposed platform, to our knowledge, is the first demonstration of a portable cell counter that integrates bright-field and fluorescence optics and is capable of on-chip sample preparation. These unique features make the proposed counting platform best suited for rapid counting of blood cells in CSF that easily degrade ex vivo. In addition, the advantages make the platform readily applicable in resource-limited settings in, for example, developing countries. Further investigations with clinical CSF samples are required to demonstrate the clinical utility of the counting platform for diagnosing pathological conditions of the CNS such as meningitis, multiple sclerosis, hemorrhages, and tumors [1,2,3,4,5]. In addition, fully automated analysis can be achieved by integrating a motorized positioning stage to scan a large area of the counting chamber with a built-in circuit board for embedded cell imaging and counting within the counting platform.

## 4. Conclusions

In summary, we developed a portable cell counting platform to detect low blood-cell concentrations in a simple, rapid, sensitive, and highly effective manner, and validated our platform by quantifying low blood cell counts from CSF samples. The platform incorporates a reagent-coated counting chamber that obviates the need for off-chip, manual sample preparation, and a miniaturized microscope for simple, portable bright-field and fluorescence cell imaging. These unique features make the counting platform attractive for clinical applications that require rapid, on-site cell analysis. On the basis of these advantages, we believe that our platform can be easily used in various clinical applications.

## Supplementary Materials

The following are available online at http://www.mdpi.com/1424-8220/18/4/1124/s1. Figure S1: Layout of the internal optical components of the miniaturized microscope with geometrical dimensions. Figure S2: Cell counting procedures by the custom Matlab algorithm that detects circular objects after image processing. Figure S3: Custom Matlab GUI and cell counting program. Blood cells in bright-field and fluorescence images were overlaid with circle symbols for visualization. Figure S4: Background images in the fluorescence mode of the miniaturized microscopy with and without the long-pass filter. Without the filter, the phosphor of the white LED can be unintentionally illuminated by UV light that significantly increases background brightness level. Figure S5: Background fluorescence level over time at *c*_d_ = 750 μM. Error bars: s.d. (*n* = 10).
