# Supplementary material for: On-Chip Cell Staining and Counting Platform for the Rapid Detection of Blood Cells in Cerebrospinal Fluid
Source: Sensors (Basel). 2018 Apr 7;18(4):1124. doi: 10.3390/s18041124 (PMC5948756; doi:10.3390/s18041124)
Supplement: Supplementary file 1 [file sensors-18-01124-s001.doc]

**Supporting Information for Sensors**

On-Chip Cell Staining and Counting Platform for the Rapid Detection of Blood Cells in
Cerebrospinal Fluid

Yujin Lee ^†^, Byeongyeon Kim ^†^ and Sungyoung Choi *

**1. Supporting Figures**


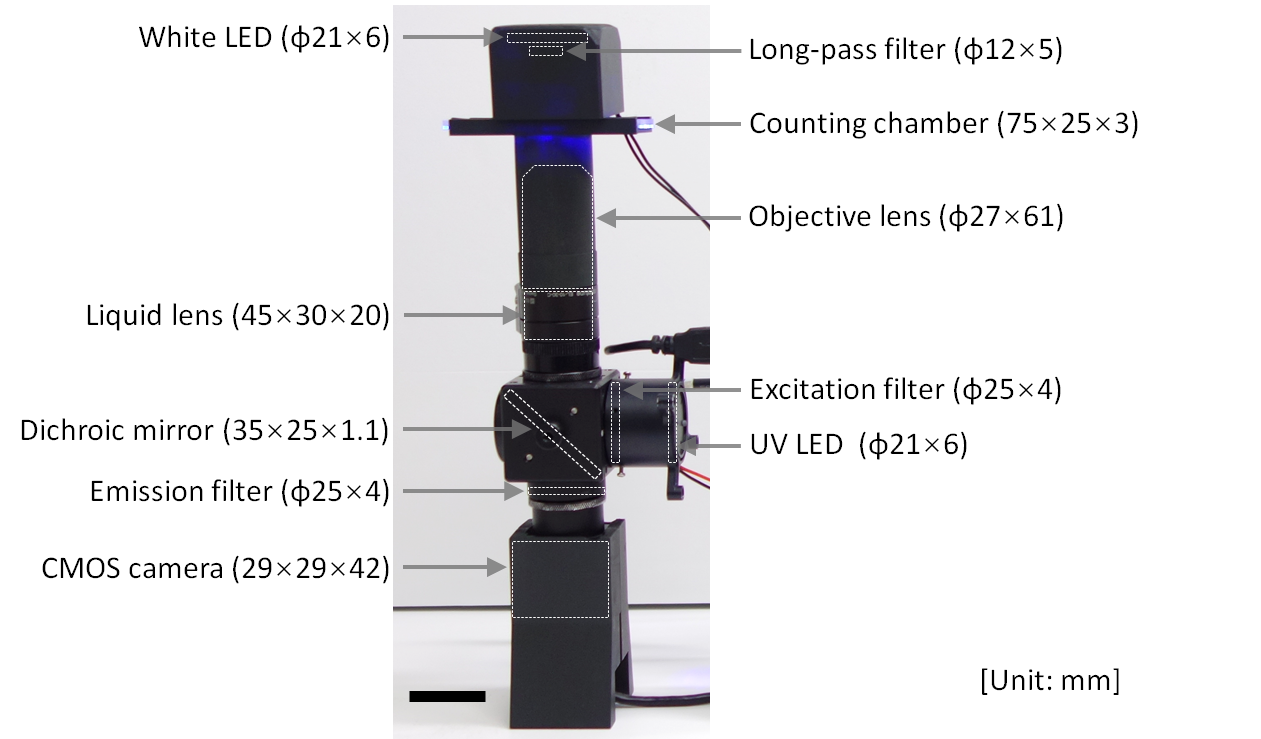


**Figure S1:** Layout of the internal optical components of the miniaturized microscope with geometrical dimensions. Scale bar, 3 cm.

**
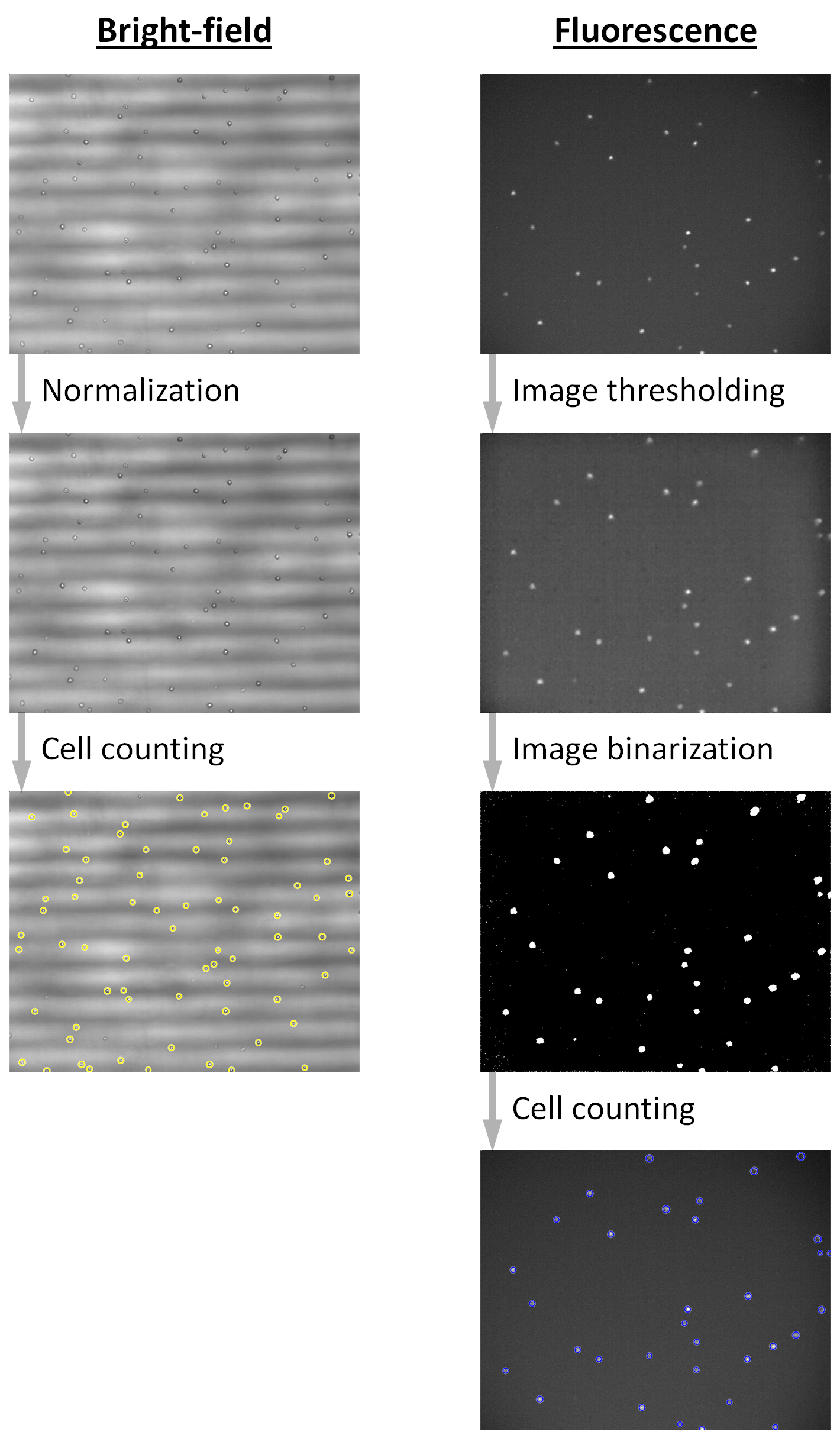
**

**Figure S2:** Cell counting procedures by the custom Matlab algorithm that detects circular objects after image processing.


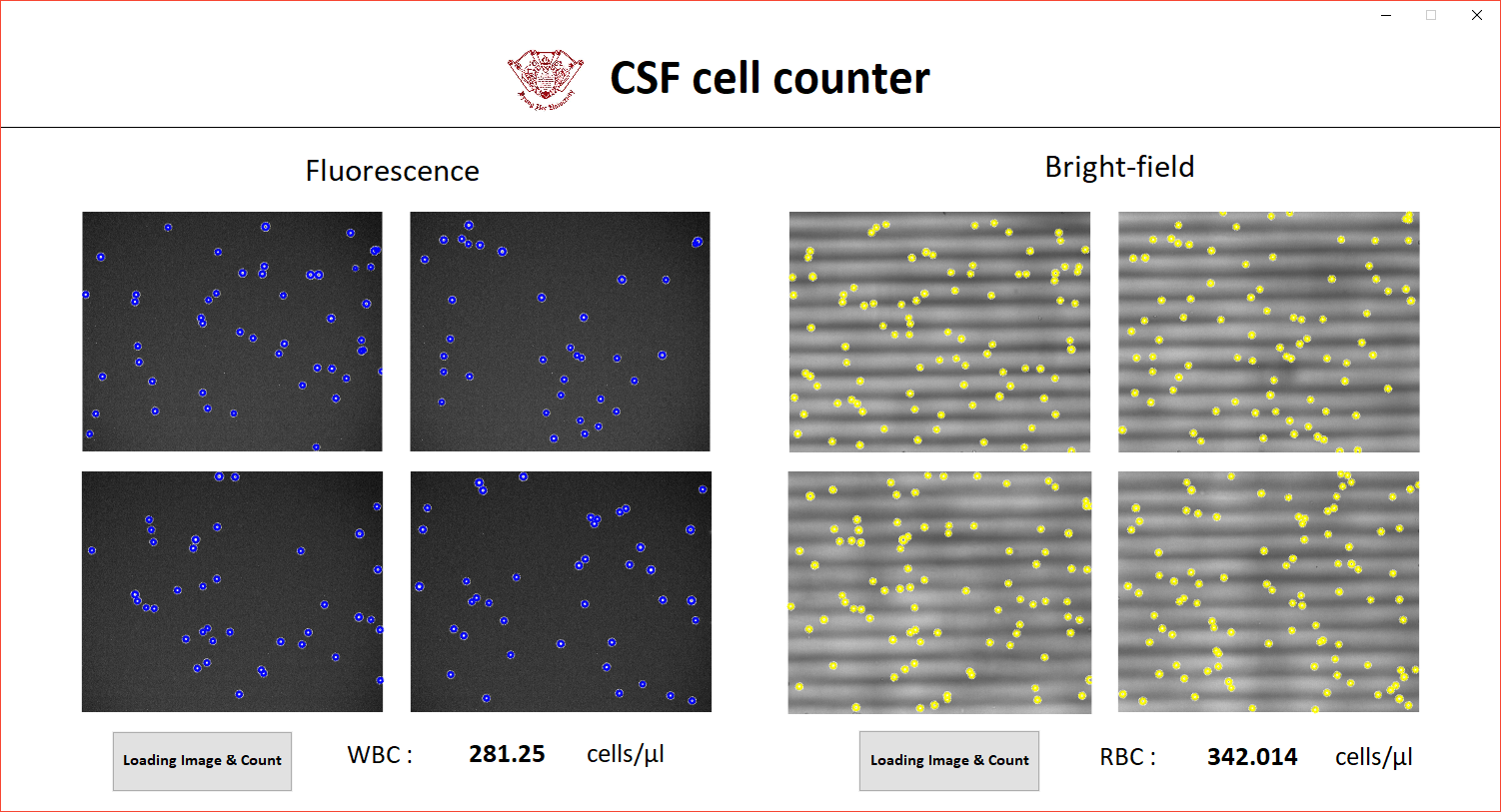


**Figure S3:** Custom Matlab GUI and cell counting program. Blood cells in bright-field and fluorescence images were overlaid with circle symbols for visualization.

**
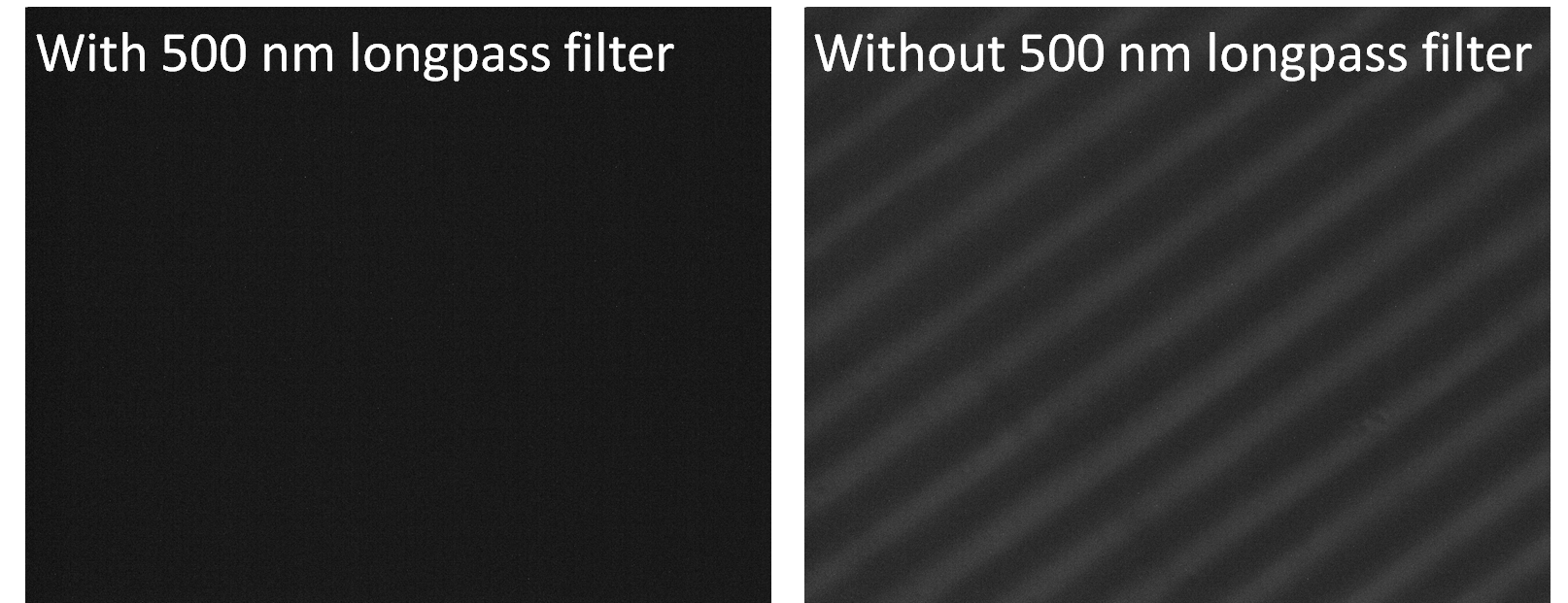
**

**Figure S4:** Background images in the fluorescence mode of the miniaturized microscopy with and without the long-pass filter. Without the filter, the phosphor of the white LED can be unintentionally illuminated by UV light that significantly increases background brightness level.

**
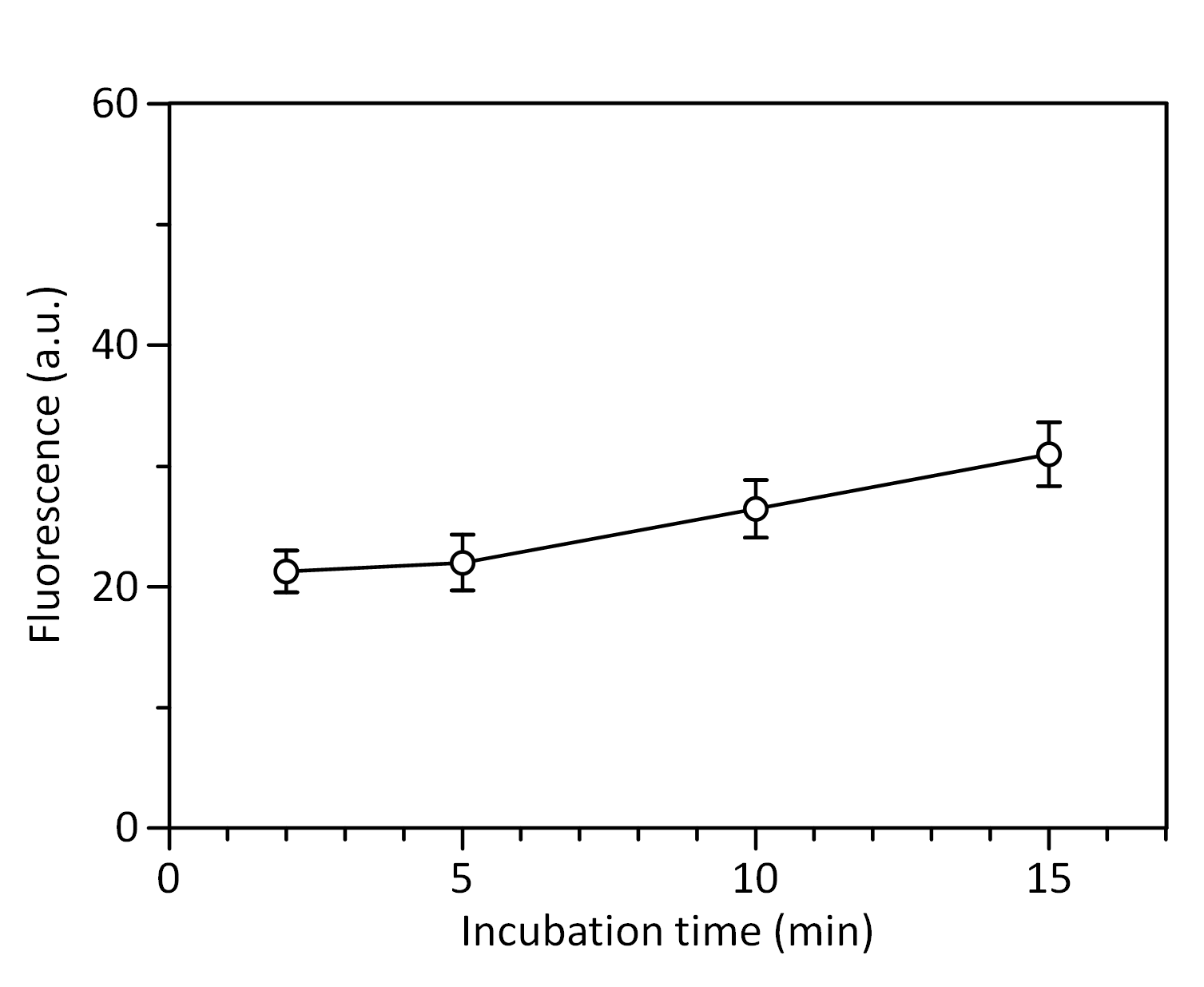
**

**Figure S5:** Background fluorescence level over time at *c*_d_ = 750 µM. Error bars: s.d. (*n* = 10).
